# Spatially explicit estimation of heat stress-related impacts of climate change on the milk production of dairy cows in the United Kingdom

**DOI:** 10.1371/journal.pone.0197076

**Published:** 2018-05-08

**Authors:** Nándor Fodor, Andreas Foskolos, Cairistiona F. E. Topp, Jon M. Moorby, László Pásztor, Christine H. Foyer

**Affiliations:** 1 Centre for Plant Sciences, Faculty of Biological Sciences, University of Leeds, Leeds, United Kingdom; 2 Agricultural Institute, Centre for Agricultural Research, Hungarian Academy of Sciences, Martonvásár, Hungary; 3 Institute of Biological, Environmental and Rural Sciences, Aberystwyth University, Aberystwyth, United Kingdom; 4 Crop and Soil Systems, Scotland's Rural College, Edinburgh, United Kingdom; 5 Institute for Soil Sciences and Agricultural Chemistry, Centre for Agricultural Research, Hungarian Academy of Sciences, Budapest, Hungary; University of Illinois, UNITED STATES

## Abstract

Dairy farming is one the most important sectors of United Kingdom (UK) agriculture. It faces major challenges due to climate change, which will have direct impacts on dairy cows as a result of heat stress. In the absence of adaptations, this could potentially lead to considerable milk loss. Using an 11-member climate projection ensemble, as well as an ensemble of 18 milk loss estimation methods, temporal changes in milk production of UK dairy cows were estimated for the 21st century at a 25 km resolution in a spatially-explicit way. While increases in UK temperatures are projected to lead to relatively low average annual milk losses, even for southern UK regions (<180 kg/cow), the ‘hottest’ 25×25 km grid cell in the hottest year in the 2090s, showed an annual milk loss exceeding 1300 kg/cow. This figure represents approximately 17% of the potential milk production of today’s average cow. Despite the potential considerable inter-annual variability of annual milk loss, as well as the large differences between the climate projections, the variety of calculation methods is likely to introduce even greater uncertainty into milk loss estimations. To address this issue, a novel, more biologically-appropriate mechanism of estimating milk loss is proposed that provides more realistic future projections. We conclude that South West England is the region most vulnerable to climate change economically, because it is characterised by a high dairy herd density and therefore potentially high heat stress-related milk loss. In the absence of mitigation measures, estimated heat stress-related annual income loss for this region by the end of this century may reach £13.4M in average years and £33.8M in extreme years.

## Introduction

Global consumption of milk is increasing in most parts of the world, driven by population and income growth, urbanization and changes in diets [1]. The UK has approximately 1.6 million dairy cows, producing about 14.6 billion litres of milk per year, making it the 10th largest milk producing country in the world. The value of UK milk production is around £4.6 billion per year, approximately 18% of gross agricultural economic output [2]. The average yield per dairy cow is over 7500 litres per annum [2].Like other agricultural sectors, milk production is influenced by the weather and climate. These factors determine what feed crops can be grown, and the availability of grass for grazing. A large proportion of UK dairy farming is based on cows grazing pastures for approximately six months of the year [3]. During the grazing period, dairy cows are more exposed to environmental factors and are thus likely to be more vulnerable to climate change than cows that are housed, especially if we consider that cooling devices can be used as a relief for cattle.

Projected changes in climate will directly impact the dairy cow, mainly as a result of heat stress, but also through the indirect effects climate change will have on pasture yield and quality, and the length of the growing and grazing season [4,5]. Farm animals have specific thermoneutral zones, which are the ranges of ambient temperatures in which body heat production is in equilibrium with body heat loss, when there is no need for additional warming (e.g. shivering) or cooling (e.g. sweating and panting) mechanisms or behaviours (e.g. seeking shelter). Abiotic factors that affect these are relative humidity (RH), wind speed and the intensity of solar radiation. Ambient temperatures (T) higher than the upper critical T of the thermoneutral zone will result in heat stress [6], leading to a net decrease in milk production in cows [7] and thus milk loss from dairy farms [8,9]. The need to predict both heat stress and corresponding milk losses led to the development of the temperature humidity index (THI), which combines effects of T and RH associated with the level of thermal stress. An animal is considered to be heat stressed with THI above specific thresholds (THI_thr_). Several THI calculation methods and THI_thr_ have been proposed in the literature [7–12] but there is no single standard procedure for calculating THI from T and RH data. Once a THI method is defined, empirical equations can be used to quantify the impact of heat stress on milk yield reductions.

As heat stress is likely to be a direct effect of climate change on dairy cows, the overall aim of the present study was to apply a modular approach to investigate the potential outcomes. In the first analysis, we considered only the impact of heat stress on milk losses from dairy cows, assuming no mitigation measures are taken. We recognise that other factors, such as cow fertility, disease and mortality rate [13] may also be affected by future heat episodes, in addition to the impact of these on general animal comfort and welfare [14], but these will be assessed in later studies. The specific objectives of this study were: 1) to predict future changes of heat stress-related milk loss of dairy cows in the UK in a spatially-explicit manner, 2) to estimate the uncertainty associated with calculated milk losses, 3) to project the possible economic consequences of milk loss due to heat stress, and 4) to assess differences between milk loss calculation methods.

## Materials and methods

### Climatic data

In the framework of the UKCP09 project [15], an 11-member data ensemble was created using 11 variants of a regional climate model (RCM, called HadRM3), based on the medium emission scenario (A1B) with data produced on a daily time scale at a 25 km spatial resolution [16]. The spatially coherent projections (SCPs) were generated by applying scaling factors to the RCM data, in a way that the changes in the SCP ensemble were linearly related to changes in global temperature. A time scaling method [17] was used to incorporate the uncertainty in global temperature from emission scenario, carbon cycle, sulphur cycle and ocean physics, into the RCM data, making the spread of the scaled RCM data consistent with the overall spread in the probabilistic (General Circulation Model based) projections [15]. The SCP ensemble was designed to be used for trend analysis as the RCM provides continuous projections for the 1950–2100 period. The SCPs used in this study explore an even wider range of climate change than the General Circulation Model driven RCM projections of the UKCP09 but still include 11 equally plausible projections of future climate conditions [16]. The grid of climate projections covers the inland territory of the UK with 440 25×25 km cells. For each grid cell, 11 different series of daily maximum and minimum temperature (T_max_ and T_min_) as well as average relative humidity (RH_mean_) data for the 2000–2100 period were used according to the 11-member SCPs.

Heat waves (frequency and length) were the focus of particular attention in these climate projections because this information is required for milk loss methods (model M5 and M6 in Table 1) that were firstly introduced in this study. A heat wave is defined as a period when the daily maximum temperature exceeds the 90th percentile of a reference distribution (years between 1980 and 2009) for at least 3 consecutive days [18].

**Table 1:** Summary of the THI-based milk loss estimation models.

#	THI calculation	Milk Loss (ML) equation	Time step	Reference for THI method	Reference for ML method
**M1**	THI = T + 0.36×T_dew_ + 41.2	ML = 0.0695×(THI_max_−THI_thr_)^2^×D	sub-daily	[21]	[8]
**M2**	THI = 1.8×T+32–(0.55–0.0055×RH)×(1.8×T– 26)	ML = 0.0695×(THI_max_−THI_thr_)^2^×D	sub-daily	[22]	[8]
**M3**	THI = T + 0.36×T_dew_ + 41.2	ML = max(THI−THI_thr_, 0)×0.37	daily	[21]	[9]
**M4**	THI = 1.8×T+32–(0.55–0.0055×RH)×(1.8×T– 26)	ML = max(THI−THI_thr_, 0)×0.39	daily	[22]	[9]
**M5**	M1 on heat wave days M3 on non heat wave days	M1 on heat wave days M3 on non heat wave days	mixed	[21]	[8,9]
**M6**	M2 on heat wave days M4 on non heat wave days	M2 on heat wave days M4 on non heat wave days	mixed	[22]	[8,9]

T, T_dew_, RH, THI_max_ and THI_thr_ denote temperature [°C], dew point temperature [°C], daily maximum of THI [] and the threshold THI [], respectively. D denotes the THI load, the duration of time the cows are experiencing heat stress in a day. T, T_dew_, and RH denote daily and hourly averages in case of daily and sub-daily models, respectively.

### Milk loss estimation methods

The daily milk loss values (kg/cow) were calculated for each grid cell by using six methods (2 sub-daily step, 2 daily step, and 2 mixed) described in Table 1 and in the supplemental material. Daily step methods use only daily values of meteorological parameters (e.g. mean relative humidity) while sub-daily step methods take the diurnal changes of meteorological parameters into account. Sub-daily climatic data were produced from the daily values by postulating an idealised sinusoidal diurnal course of the climatic variables [8]. All the investigated models incorporate a combination of THI and milk loss equations. However, for M5 and M6 (Table 1) a mixed formula was used to account for the capacity of dairy cows to avoid heat stress in shorter periods of heat stresses risk representing a more biologically appropriate way of heat stress related milk loss estimation [19,20]. These two models include a sub-daily step milk loss equation for days of heat waves and a daily step milk loss method on other days.

In European studies, the THI threshold (THI_thr_) used to calculate the risk of heat stress varies with the production system, with values ranging from 60 [11,7] to 70 [23]. For high yielding dairy cows, Zimbelman et al (2009) proposed a THI_thr_ of 68 [24]. In the USA, typically the threshold is set at 72 [8–10]. Most of these studies used T_max_ and RH_min_ [11,7], but in others the average daily THI of an hourly calculated THI [11] or the THI load [8] were used. Therefore, models M1-6 were combined with THI_thr_ values of 68, 70 and 72 resulting in 18 different investigated models.

These models were used to estimate milk loss in each grid cell without taking into account the type of dairy farming system (at pasture vs indoors). It was assumed that temperature and relative humidity were the same for all systems, and that no mitigation practices were implemented. We also assumed that cattle were not significantly different from the current UK breed types, even though breeding for heat stress tolerance is one of the proposed measures to mitigate effects of climate change on dairy farms [25].

### Assessment of the impact of climate change and the uncertainty of milk loss projection

The annual milk loss per cow (AML, kg/cow/y) value was used to assess the projected impact of climate change on milk production and was calculated using each model as the summation of predicted daily losses for each year. The 11 climate projections and the 18 calculation methods resulted in 1980 AML values for every grid cell for every decade from the 2010s to the 2090s. The uncertainty of the calculated AML values was characterised with the coefficient of variation (CV, standard deviation (SD) divided by the mean) for each grid cell. The uncertainty of the AML figures originates from three major sources: 1) Year effect: caused by the interannual variability of temperature and humidity patterns within a decade; 2) Climate Projection effect: caused by the differences in the climate model projections; 3) Method effect: caused by the differences in the milk loss calculation methods. The contribution of these three factors to the overall uncertainty of AML was quantified as follows: 1) Year effect: for every year the average of AMLs obtained for each climate projection and method combination was calculated (average of 198 values). Then, the coefficient of variation was calculated across the years (CV of 10 values). 2) Climate Projection effect: for every climate projection the average of AMLs obtained for each year and method combination was calculated (average of 180 values). Then, the coefficient of variation was calculated across the climate projections (CV of 11 values). 3) Method effect: for every method the average of AMLs obtained for each year and climate projection combination was calculated (average of 110 values). Then, the coefficient of variation was calculated across the methods (CV of 18 values).

In order to obtain comparable CV values that are calculated from samples having considerably different sizes (10 or 11 versus 18) the jackknife resampling method [26] was applied. In case of calculating the CV indicating the Method effect 10 member sub-sets were selected from the 18 member base set in all possible ways resulting in C_18,10_ = 18!/10!/8! = 43,758 different sub-sets. The CV values of each of the sub-sets were calculated and the mean of the 43,758 member distribution was used as an indicator for the Method effect.

A detailed assessment of the sub-daily (M1-2) and daily (M3-4) methods was performed to reveal the most important cause of the differences between the results of the two method types. This analysis was carried out using all three THI thresholds (68, 70 and 72) but only the results obtained with THI_thr_ = 70 were presented for a selected grid cell. The number of days affected by heat stress (THI_d_ > THI_thr_) as well as the number of days characterised by THI_max_ > THI_thr_ and THI_d_ < THI_thr_ was determined for each grid cell and for every year of the 2010–2100 period. The latter indicates the days when the daily step methods predict no heat-stress and no milk loss while sub-daily step methods predict a considerable milk loss. In general, conditions when THId > THIthr represent greater severe heat stress potential than at other times.

The characteristics of trends in AML and number of heat stress days from years 2010–2100 were investigated by regression analysis in STATISTICA 12.0 [27]. An example grid cell in South-East England (centroid: 51.0°N, 0.7°W) was selected to represent an area that climate change is projected to cause considerable changes compared to the baseline, and detailed temporal changes of AML and number of heat stress days were calculated for this cell. Linear and exponential trends of these data were considered, with the best regression model fits being determined using the coefficient of determination (R^2^) and the normalised root-mean-square error (NRMSE = 100×RMSE/MEAN, where RMSE and MEAN were the root-mean-square error and the average of the calculated values, respectively) of the fitted curves. The curve type providing the better statistical indicators was used to characterise the trend in question. The significance of the difference between the milk loss projections was tested using Mann–Whitney U test in STATISTICA.

### Economic consequence of milk loss due to heat stress

The financial aspect of heat stress related milk loss was estimated for each of the NUTS-1 regions of the UK. The Nomenclature of Territorial Units for Statistics (NUTS) system is a geocode standard for referencing the subdivisions of EU member countries for statistical purposes [28]. The regional annual milk loss (RAML) values (kg/cow) were calculated by aggregating the AML values of the grid cells belonging to the particular NUTS1 region. According to a DairyCo report [29], approximately 81% of a herd is potentially affected by heat stress as its lactating period overlaps with the summer months. The average herd sizes (AHS) of the NUTS1 regions were retrieved from the AHDB database [30]. Numbers of cows per dairy farm have steadily increased during the past 20 years with a rate of 3.5 cow/y across the whole of the UK, and thus the income loss (IL, £/y) of a typical dairy farm (having an AHS, at pasture) was calculated for each NUTS-1 region according to two scenarios. Scenario_1 postulated no more centralisation of herds, thus stagnating AHS (constant AHS) and Scenario_2 postulated a continuous growth of AHS with a rate that was observed in the past two decades (increasing AHS). As the farm-gate milk price fluctuates around £0.3 per litre and does not show any specific long-term trend [2], the income loss of a typical UK dairy farm can be estimated with the following equation:

IL=RAML×AHS×0.81×0.3 (1)

Income losses were calculated for average years (when RAML is the average of AMLs) as well as for extreme years (when RAML equates to the 90th percentile of AMLs).

## Results

### Spatial and temporal changes of heat stress and milk loss

**Fig 1:**
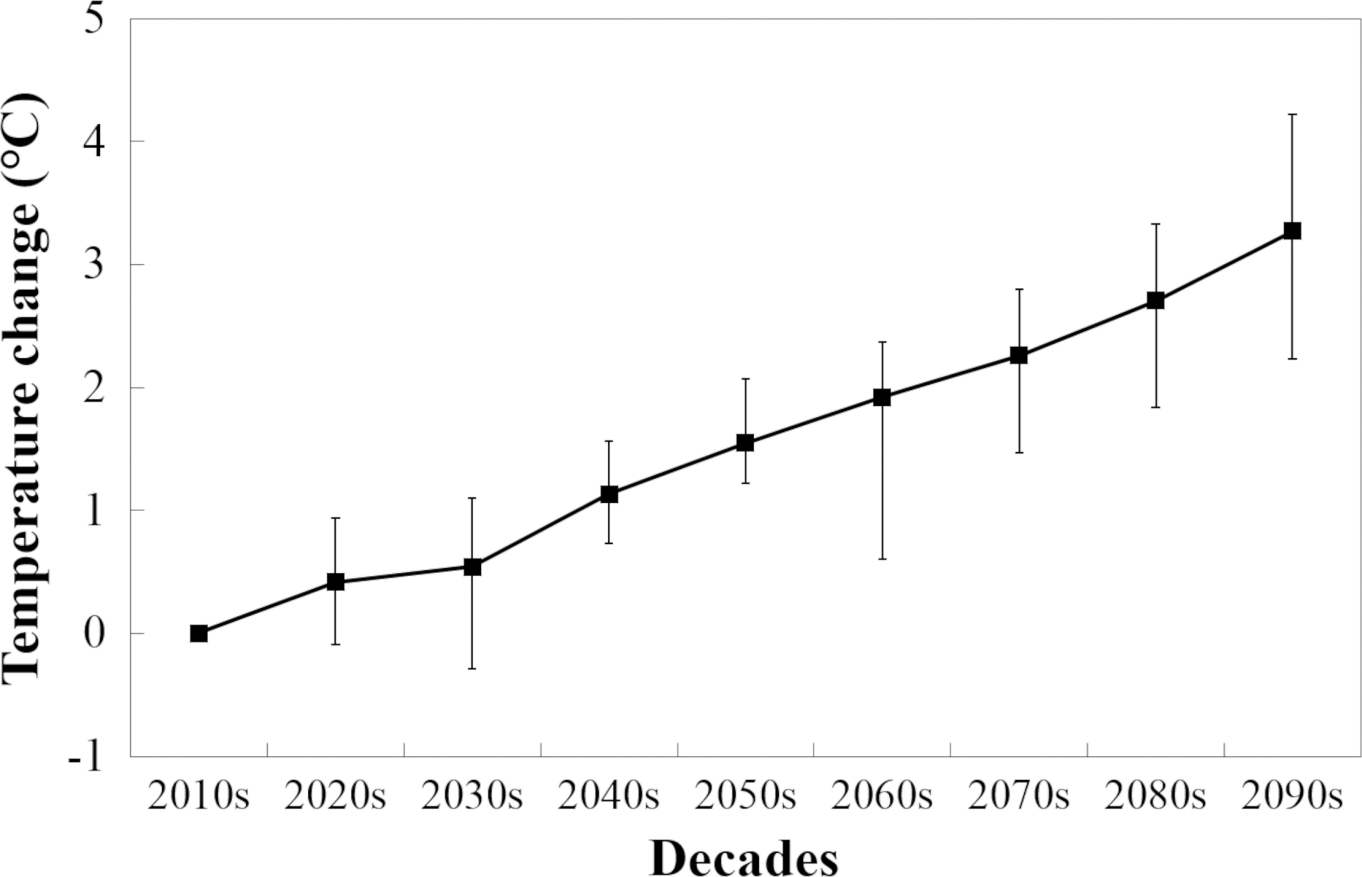
Changes in mean daily temperature according to the 11-member spatially-coherent RCM projection ensemble for the summer period (April-September) in the UK. The vertical bars denote the range between the minimum and the maximum values predicted by the 11 climate projections. Baseline period: 2010s.

**Fig 2:**
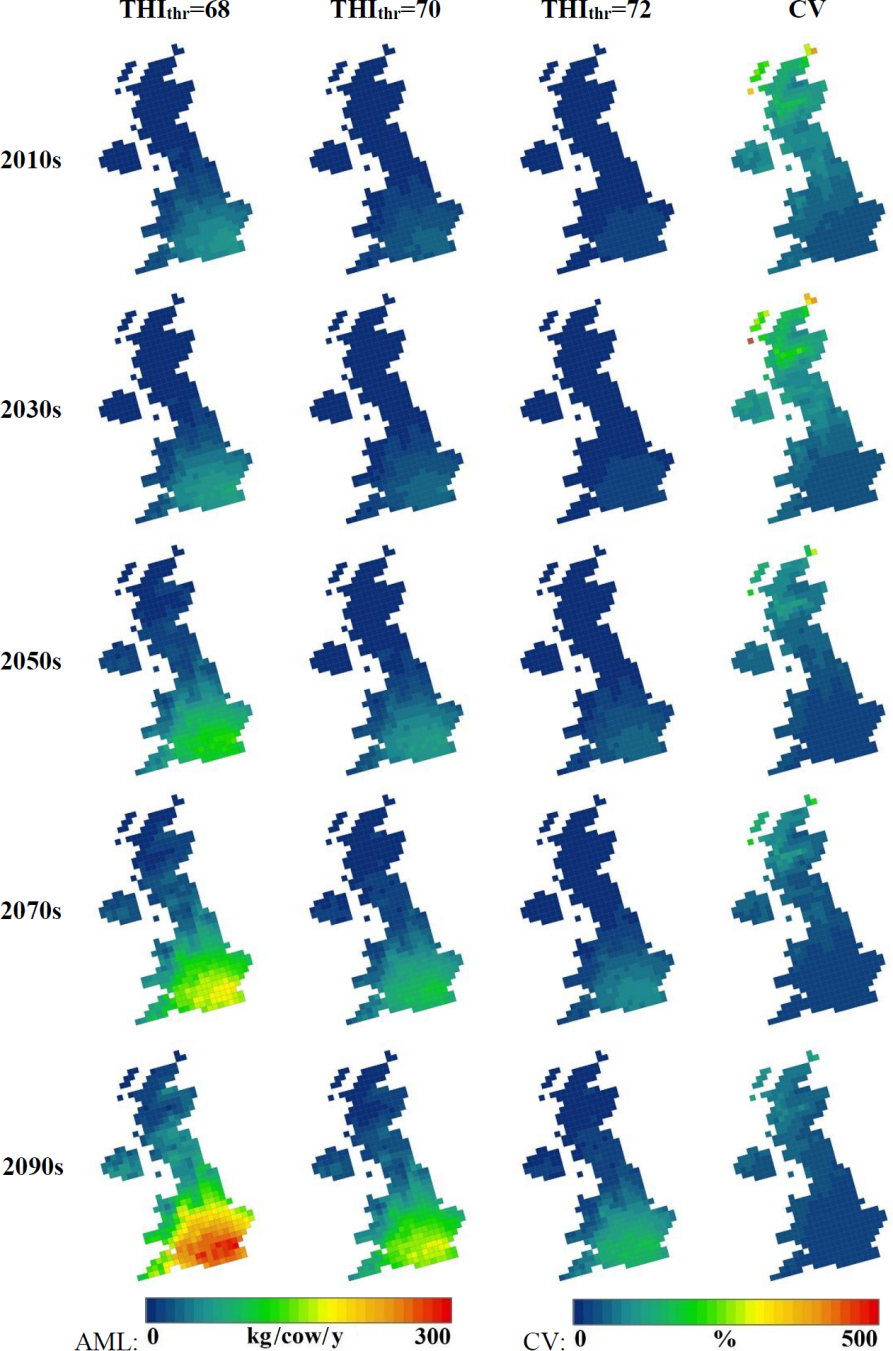
Maps of annual milk loss for each THI_thr_ and the CV for the 2010s, 2030s, 2050s, 2070s and 2090s in the UK. CV of 1980 values per each cell.

Fig 1. shows the trends of temperature changes in the UK for the summer period (April-September) defined by the SCPs. As a result of these, the AML per cow values varied between regions across the UK. The average current AML was calculated to be around 1 kg/cow in the north of the UK while in the south it may reach 40 kg/cow. This difference is expected to increase under future climate scenarios (Fig 2). By the end of the century, dairy cattle in large portions of Scotland and Northern Ireland will experience the same level of heat stress as cattle in southern-England today. In South East England the average AML was projected to exceed 170 kg/cow based on the average of the 18 investigated methods. The projected AML values were highly dependent on the selected threshold THI. Changing THI_thr_ from 72 to 68 resulted in an increase of projected AML from 80 to 320 kg/cow for the most affected regions in the South (Fig 2). The average AML predicted for the 2090s was relatively low (2.4% of the annual milk yield) even for the South East England region. On the other hand, an unlikely extreme event (once in every 10 years) would mean the maximum AML may be close to 600 kg/cow (8.0% of the annual milk yield) (Table 2). At the most extreme, in the ‘hottest’ 25 km grid cells (around the Greater London area) in the hottest years, AML may exceed 1300 kg/cow which is 17% of the potential current mean milk production. The uncertainty (CV) of the AML was lower in the South than in the North due to the fact that the AML values were considerably lower in the North. The uncertainty is expected to decline slowly during the century but the North-South difference will remain constant according to the projections. The decrease in the coefficient of variation was the result of the average of AMLs increasing more rapidly than their standard deviation.

**Table 2:** Statistical description of heat stress days and milk loss values in the 11 UK NUTS-1 regions.

NUTS-1 region		Heat stress days		Milk loss (kg/cow/y)				
		Day1	Day2	Max	Mean	StDev	Median	90th percentile
**Scotland**	**2010s**	0.6	6.4	191.0	1.8	6.4	0.0	4.7
	**2050s**	2.0	13.9	240.4	5.0	11.9	0.8	13.9
	**2090s**	6.6	21.3	650.2	15.6	33.9	3.6	41.5
**Northern Ireland**	**2010s**	1.0	10.0	164.4	2.7	7.7	0.2	6.9
	**2050s**	3.5	20.8	212.3	7.8	16.3	2.4	21.4
	**2090s**	11.6	32.5	515.7	25.8	43.0	10.6	67.7
**North West England**	**2010s**	2.5	14.8	310.6	7.1	16.9	1.4	19.3
	**2050s**	7.3	28.0	428.6	19.4	34.1	6.9	53.5
	**2090s**	17.2	36.7	900.3	48.1	74.1	20.4	126.5
**North East England**	**2010s**	2.0	15.4	153.6	5.5	12.0	1.0	14.9
	**2050s**	7.0	29.5	275.2	16.0	25.8	6.0	43.7
	**2090s**	16.9	38.4	546.6	42.1	59.0	19.7	109.0
**Yorkshire and The Humber**	**2010s**	3.4	21.5	217.5	9.2	17.9	2.4	27.0
	**2050s**	10.4	37.2	387.9	26.2	40.9	11.5	73.2
	**2090s**	22.6	45.2	757.6	61.2	80.4	31.0	159.0
**West Midlands**	**2010s**	7.4	32.6	391.9	21.7	34.5	7.6	59.3
	**2050s**	19.7	47.8	605.5	56.5	75.1	29.4	154.4
	**2090s**	35.8	52.4	1129.5	112.9	137.1	64.9	297.7
**East Midlands**	**2010s**	7.6	34.2	363.8	22.2	35.4	8.2	60.0
	**2050s**	19.9	48.6	628.3	57.6	75.8	29.5	153.7
	**2090s**	35.8	52.7	1116.8	111.3	132.1	65.4	292.1
**Wales**	**2010s**	4.4	18.7	424.5	11.4	24.2	2.9	31.3
	**2050s**	12.0	33.5	613.6	32.6	51.6	13.0	83.0
	**2090s**	24.8	40.6	1222.7	70.5	105.4	33.3	187.6
**East of England**	**2010s**	10.7	40.2	379.7	29.8	43.4	13.3	79.2
	**2050s**	26.3	51.4	727.0	75.7	88.5	40.4	194.6
	**2090s**	43.8	52.7	1257.8	136.4	149.4	81.8	348.1
**South West England**	**2010s**	8.6	32.0	459.0	22.9	39.0	7.8	62.6
	**2050s**	23.2	46.0	656.1	63.4	83.5	31.7	160.7
	**2090s**	41.3	49.5	1270.2	130.6	153.3	71.1	329.5
**South East England**	**2010s**	13.6	42.5	469.9	37.9	50.8	17.3	101.3
	**2050s**	31.8	51.7	741.8	92.8	108.6	52.6	235.4
	**2090s**	51.0	52.0	1310.3	171.9	178.1	105.7	432.9

Day1: number of days when THI_d_>70; Day2: number of days when THI_d_<70 but THI_max_>70. Greater London was merged with the SE England when statistics were calculated. Both the minimum and the 10^th^ percentile are practically zero for all the NUTS-1 regions in the UK.

### Uncertainty of milk loss projection

Except for the 2030s, the CV associated with the milk loss calculation method effect was consistently higher than that associated with methods of calculating climate projection and inter-annual variability for the investigated future time slots (Table 3). Despite the considerable inter-annual variability of the AML, as well as the large differences between the climate projections (Fig 1), the arbitrary selection of a milk loss calculation method may introduce similar, or even greater, uncertainty in milk loss projections. Toward the end of this century, the effect of all three calculation factors gradually decreased, which reflects the fact that the average of AMLs increases exponentially. The UK average of the AMLs in the 2090s is projected to be 4.6 times greater than the baseline, as a result of more frequent and more pronounced periods of heat stress. However, the SD of the AML values was calculated to increase linearly, so that the UK average of the SDs in the 2090s was 3.0 times greater than that of the baseline.

**Table 3:** Uncertainty (measured by CV, %) of milk loss projections originating from different sources.

	Decades				
	2010s	2030s	2050s	2070s	2090s
**Year effect**	51.4	77.3	42.0	31.0	26.8
**CP effect**	54.6	68.3	38.6	38.8	24.7
**Method effect**	54.3	63.2	45.9	41.0	35.9

Sources of uncertainty: inter-annual variability (Year effect); differences between the climate projections (CP effect); different milk loss estimation methods (Method effect).

### Assessing the milk loss estimation methods

**Fig 3:**
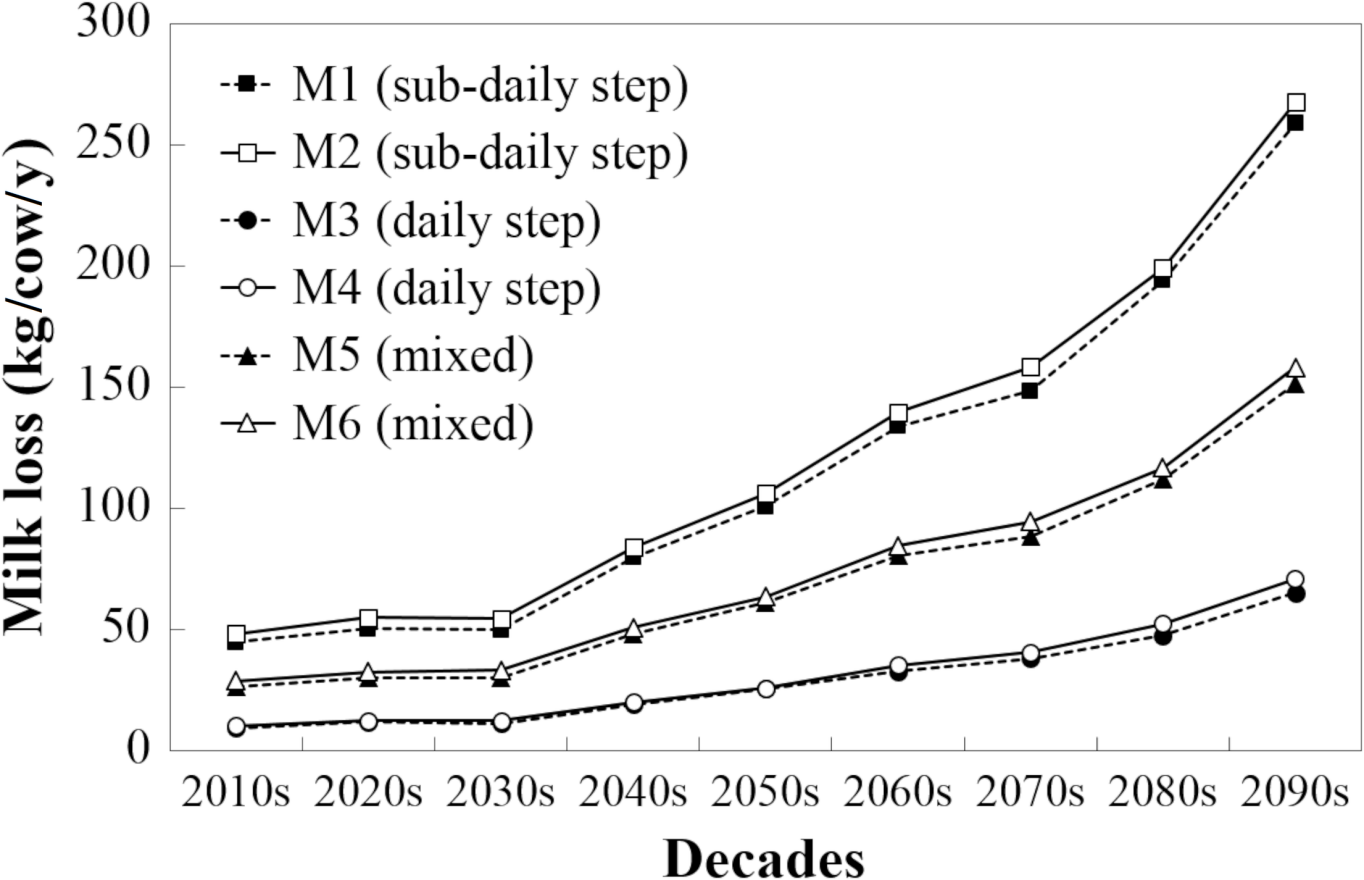
Changes of average annual milk loss values calculated with six different milk loss methods (THI_thr_ = 70) for a 25×25 km grid cell in South-East England (centroid: 51.0°N, 0.7°W). Form of the fitted exponential curve: AML = a×e^b×(y-2010)^; M1-2: a = 27.86, b = 0.0234, SE_a_ = 1.68, SE_b_ = 0.00074, R^2^ = 0.989, NRMSE = 7.4%; M3-4: a = 6.13, b = 0.0251, SE_a_ = 0.456, SE_b_ = 0.0009, R^2^ = 0.986, NRMSE = 6.6%; M5-6: a = 17.19, b = 0.0229, SE_a_ = 1.11, SE_b_ = 0.00079, R^2^ = 0.986; NRMSE = 6.8%. U test showed significant difference (P < 0.001) between the milk loss calculation methods: M1-2 different from M3-4 and both pairs different from M5-6.

Both the daily and the sub-daily step methods showed an exponential increase in AMLs. The sub-daily step methods (M1-2), however, project a much more substantial rise in AML. Fig 3 presents an example of the changes of AML throughout the investigated period for the selected grid cell in South-East England.

**Fig 4:**
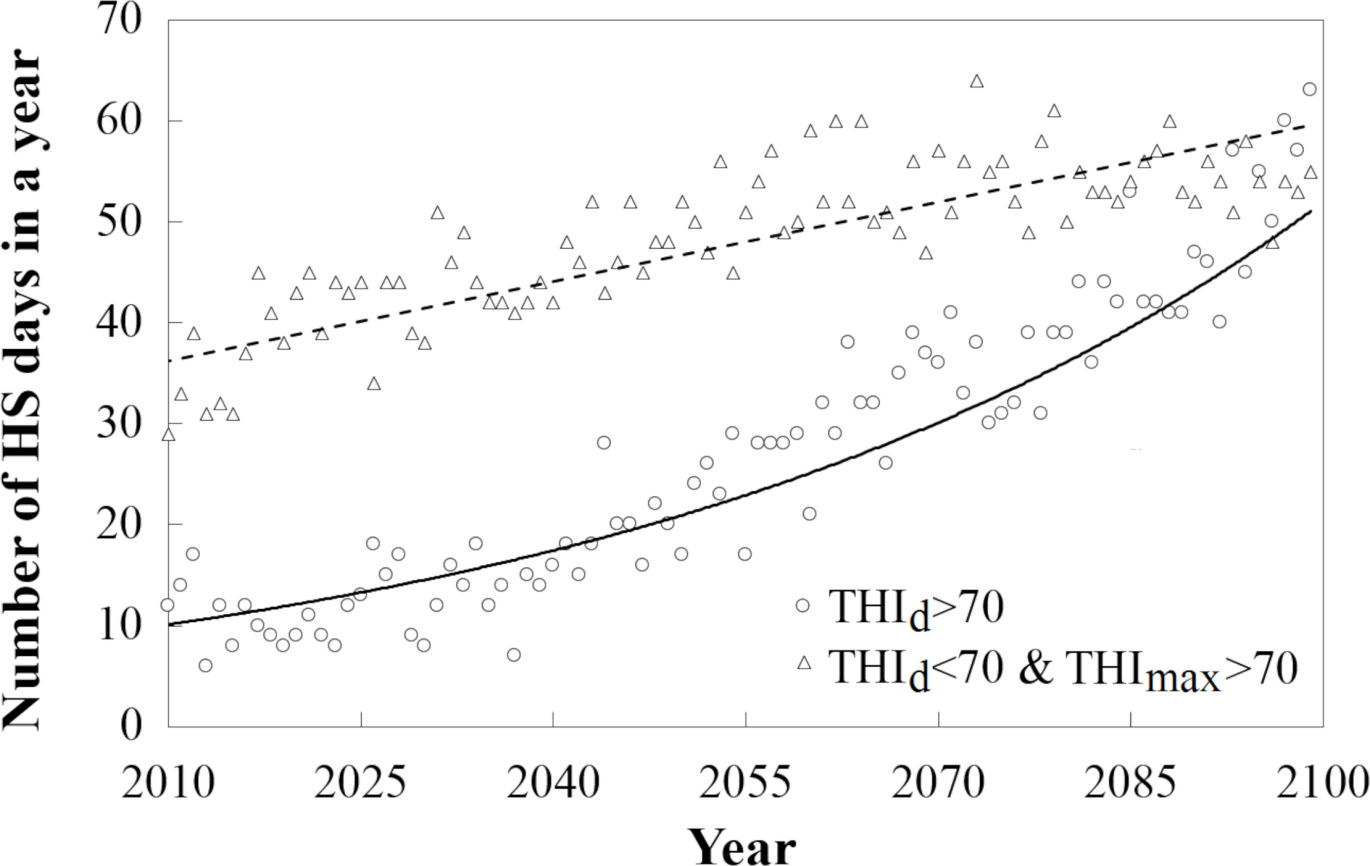
Changes in the number of days affected by heat stress (HS) calculated with the St-Pierre method (see M2 method description; THI_thr_ = 70) for a 25×25 km grid cell in South-East England (centroid: 51.0°N, 0.7°W). Triangles (and the fitted dotted linear) denote the days with heat stress that are detected only by the sub-daily methods (THI_d_<THI_thr_ and THI_max_>THI_thr_). Circles (and the fitted exponential curve) denote the days with heat stress that are detected by both the sub-daily and daily methods (THI_d_>THI_thr_). Circles: number of heat stress days = a×e^b×(y-2010)^, a = 9.69, b = 0.0199, SE_a_ = 0.523, SE_b_ = 0.00077, R^2^ = 0.91, NRMSE = 16.2%; Triangles: number of heat stress days = a×(y-2010)+b, a = 0.23, b = 38.28, SE_a_ = 0.0184, SE_b_ = 0.95, R^2^ = 0.639; NRMSE = 15.7%.

**Fig 5:**
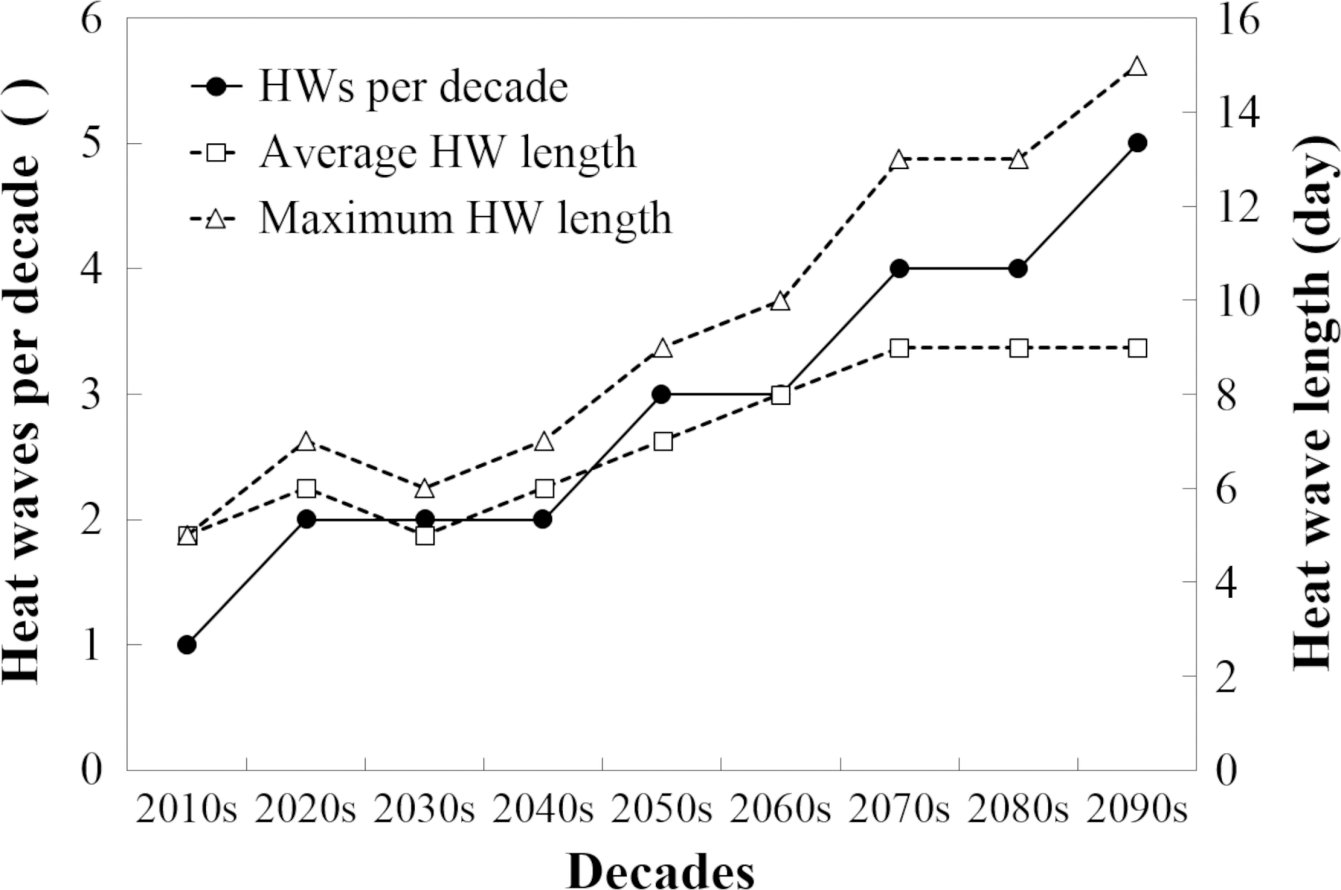
Frequency and length of heat waves (HW) in the UK. The presented values are the average of 11 UKCP09 SCP climate projections.

The exponential increase in milk loss (Fig 3) was due to the fact that the number of days affected by heat stress (THI_d_>THI_thr_) was projected to increase exponentially in the future irrespective of which method was selected to calculate THI (Fig 4). However, there is a linear increase in the number of days with heat stress that was only predicted using the sub-daily step methods (Fig 4). On days with THI_max_>THI_thr_ and THI_d_<THI_thr_, sub-daily methods suggest that milk loss could be as high as 2.9 kg/cow/d whereas the daily step methods would predict no milk loss. Since the number of these partially heat-stress affected days is projected to increase linearly (Fig 4), the difference between the sub-daily step and daily step methods is also projected to increase in the future. Currently (in the 2010s), the daily step methods would indicate that the number of heat stressed days is approximately 20% of those calculated using the sub-daily methods (Fig 4). Due to the projected increase in temperature, the difference between the daily and sub-daily methods will decline, but daily methods will still only capture around 50% of the days predicted by the sub-daily methods as heat stressed. However, the frequency and length of heat waves is predicted to increase in the UK throughout the century (Fig 5), with a subsequent potential effect on AML.

### Economic consequence of milk loss due to heat stress

Compared to current the UK average annual dairy farm business income (£80,000) the heat stress-related income loss was projected to be less than 7% even in the most affected southern UK regions towards the end of the century (Table 4). In extreme years, however, the income loss may reach as high as 18% in South East England, though the dairy cow density in this region is relatively low. South West England is the most vulnerable to climate change as this is the region which is characterised by a high dairy herd density and therefore high potential heat stress-related milk loss. Estimated heat stress-related annual income loss of the region may reach £13.4M in average years, and £33.8M in extreme years (at current values), by the end of the century if no action is taken to mitigate it.

**Table 4:** Income loss of average size dairy farms in different UK NUTS-1 regions due to heat stress (£/y) assuming no mitigation actions are taken.

NUTS-1 region	Period	Average Year		Extreme Year	
		scenario_1	scenario_2	scenario_1	scenario_2
**Scotland**	**2010s**	86	86	217	217
	**2050s**	232	401	649	1122
	**2090s**	727	1787	1936	4760
**Northern Ireland**	**2010s**	72	72	187	187
	**2050s**	210	475	578	1308
	**2090s**	696	2452	1826	6431
**North West England**	**2010s**	235	235	638	638
	**2050s**	641	1301	1768	3588
	**2090s**	1591	4867	4181	12788
**North East England**	**2010s**	183	183	494	494
	**2050s**	530	1075	1444	2930
	**2090s**	1390	4252	3602	11019
**Yorkshire and the Humber**	**2010s**	305	305	893	893
	**2050s**	867	1760	2421	4913
	**2090s**	2021	6182	5254	16070
**West Midlands**	**2010s**	717	717	1958	1958
	**2050s**	1866	3787	5103	10356
	**2090s**	3732	11415	9839	30096
**East Midlands**	**2010s**	733	733	1985	1985
	**2050s**	1905	3866	5079	10306
	**2090s**	3680	11255	9652	29525
**Wales**	**2010s**	350	350	966	966
	**2050s**	1007	2116	2560	5382
	**2090s**	2175	6970	5790	18555
**East of England**	**2010s**	984	984	2619	2619
	**2050s**	2503	5079	6430	13048
	**2090s**	4509	13792	11505	35192
**South West England**	**2010s**	758	758	2070	2070
	**2050s**	2097	4255	5310	10776
	**2090s**	4317	13205	10889	33308
**South East England**	**2010s**	1253	1253	3346	3346
	**2050s**	3068	6226	7779	15787
	**2090s**	5682	17382	14307	43763

Average year (milk loss = average of AMLs). Extreme year (milk loss = 90th percentile of AMLs). Scenario_1: no more centralisation of herds (constant AHS); Scenario_2: continuous growth of AHS with a rate that was observed in the past two decades (increasing AHS).

## Discussion

### Animal responses to heat stress

A relatively low rate of occurrence of heat stress in UK dairy cows in the current climate (2010s) was estimated by all the methods used in the present study. Similarly, Dunn et al (2014) and Hill and Wall (2015) reported an average of one day of heat stress conditions per year [23,31]. However this can increase to five days when years with heat waves are considered [23]. However, these studies failed to detect any significant milk yield reductions due to heat stress in 2003 and 2006, when strong heat waves were recorded [23,31]. Our analysis suggests that the average AML in regions with high heat stresses (e.g. South East England) is 40 kg/cow. However, this reduction was calculated from total days of heat stress conditions without taking into account the fact that these days were not consecutive. Cattle initially respond to mild heat stress by sweating, panting, drinking more, and seeking shade when possible. At higher temperatures cows reduce their feed intake, which leads to a fall in milk production. When heat stress is temporary, lasting only one or two days, it is possible that cows will not reduce their feed intake or their milk production [32]. Therefore, it is not surprising that under current UK climatic conditions there are no evident milk yield penalties even when model simulations predict small decreases. By the end of the century, the average UK daily temperature was projected to be 4°C higher than the current temperature (Fig 1). This will result in a projected increase in the number of heat stress periods across all the regions of the UK. The corresponding AML was estimated to be 105 kg/cow if no steps are taken to adapt to changing climate conditions and UK dairy cattle remain the same in terms of genetic merit and heat tolerance. Even though this cannot be categorised as severe heat stress conditions, a noteworthy AML was estimated. This estimation assumes no difference in cows kept indoors and outdoors because it is not possible to differentiate between farm types in each grid cell. However, it is well established that temperatures in dairy barns are 3 to 6°C higher than those measured outdoors [33,34], and the projected temperature and relative humidity values our calculations used were for outdoors. Thus, unless some form of indoor temperature management is adopted, actual AML may be even higher than calculated here for some farm types.

British dairy farming is heavily reliant on pasture use [35]. A number of relatively low cost adaptation measures could help minimize adverse consequences of heat stress in dairy cows. The provision of natural or artificial shade is the most efficient and inexpensive way to reduce heat accumulation from solar radiation, leading to reduced signs of heat load, rectal temperatures and hyperchloraemia [36,37]. Shade provision can lead to increased milk yield of pasture-based cows [38]. Recently, a field study was conducted to investigate the effects of the amount of shade on 8 Holstein-Friesian pasture-based herds in New Zealand for 2 consecutive summers. It was reported that providing more shade increased the proportion of animals within the herd that used this resource and reduced respiratory signs of heat load [39]. Similarly, when shade was provided to Holstein cows on pasture using young trees to support shade cloths, it tempered the effects of increased THI by reducing rectal temperature, hyperchloraemia and the regulation of liver metabolism [40]. Heat-stressed dairy cattle kept indoor increase their water intake by 22–27% [41, 42]. Although these figures maybe lower for outdoor cattle due to the availability of fresh forage with a high water content the distance between available water and the grazing area should allow at least twice daily visits by the cattle [43]. Nutritional management might also provide a cost-effective mechanism to support heat-stressed cattle. This might include supplementation of rumen-protected proteins and fats, electrolytes, and specific feed additives [44].

Although average UK temperature increases are estimated to have relatively minor impact in many regions, our analysis predicted that heat waves could lead to severe heat stress in dairy cows with projected AMLs greater than 1,200 kg/cow by the end of the century in high-risk areas. These areas are Wales, South West, South East England, and East of England, although potential total milk losses in Wales and the South West are likely to be higher than in the South East and East of England because of the higher concentrations of dairy cattle in the west of the UK [45]. This finding is in accordance with other studies that reported increased parasite risk in specific UK regions due to climate change [46]. The increased occurrence of heat waves worldwide [47] and in the UK (Fig 5) is expected to cause additional heat stress in dairy cows, as reflected to our analysis where extreme years show a correlation between a high frequency of heat waves and the maximum AML. The current frequency of 1–2 heat waves per decade may increase to 3–5 by the end of the century, which is much lower than currently observed, for example, in Italy (5 heat waves per year [48]). However, the length of a heat wave is projected to reach 8 to 15 days similar to that currently reported for Italy [48]. In such conditions, heat stress is correlated not only with increased milk loss, but also with increased cattle mortality [13,48] and culling due to reduced fertility [49]. In these cases, simple adaptation measures, such as the provision of shade, may not be sufficient to mitigate negative heat stress effects on milk production. However, current technologies used in other, hotter, parts of the world (e.g. fans and water misting) could be applied to British dairy farming, which is already changing towards intensive indoor systems [35], to care for dairy cows during these heat waves [50]. Moreover, breeding for increased heat tolerance is a potential strategy to help mitigate negative effects of increased frequency of heat waves [25]. This can be beneficial for maintaining pasture-based systems [51,52]. Even though the main strategy to date has been crossing Holstein cows with local breeds [53], genomic predictions for heat tolerance of Holstein cows have been identified suggesting that genomic selection may accelerate breeding for heat tolerance [54,55]. In addition, changing the location of farming operations is a current practice used to address economic challenges worldwide [56, 57]. Even though there is little indication that movement of dairy farming operations is a feasible strategy to decrease the risks of environmental challenges in the UK [58], the increased use of regions with little or no prediction of conditions leading to heat stress (e.g. Scotland) may provide an additional adaptation measure for UK dairy farming depending on the availability of pasture. At the end of this century, heat stress-related annual income losses of average size dairy farms in the most affected regions may vary between £2000-£6000 and £6000-£14000 in average and extreme years respectively. Armed with these figures, farmers can easily create preliminary financial plans to assess the pay-offs of possible mitigation options such as planting trees or installing shades. It is likely that the hotter UK areas will see a reduction in cattle numbers, perhaps with increases in other areas, e.g. further north or at higher altitudes, if cropping and grazing options change to become more favourable for cows.

### Uncertainty and assessment of model simulations

The variability of the AML projections was disaggregated into three major components and the uncertainty originating from, 1) the different climate projections, 2) the inter-annual variability of the weather, and 3) the different milk loss calculation methods. These were estimated by using the coefficient of variation of the AML values. Despite the considerable inter-annual variability of the AML as well as the large differences between the climate projections, the variety of the calculation methods may introduce even larger uncertainty in the milk loss projections for the future. This finding is in line with previous climate change impact assessments. Inter-annual variability was predicted to increase slightly at higher temperatures (toward the end of the century) but this effect was generally less than inter-model variability [59]. Model differences introduced more uncertainty in the climate change impact projections than the differences caused by the climate projections [60]. This finding emphasizes the importance of using multi-model ensembles in order to provide robust projections [61]. Though they investigated global maize production, Bassu et al (2014) reported that only an ensemble of at least 8–10 models was able to simulate absolute yields accurately [59]. Here we demonstrate that for the South-East of England (Fig 4) this uncertainty was introduced by the selection of the daily or sub-daily AML calculation methods, where the sub-daily methods over-predicted heat stress days. Indeed, for current South-East of England conditions, the sub-daily methods predict 39 days of heat stress, while the daily step methods estimate 9 days (Fig 4). The latter is perhaps closer to current British conditions, where heat stress is not generally considered a major issue.

In general, THI_max_ is used for the sub-daily methods whereas THI_d_ is used for the daily step methods. This methodological difference explains why there was a greater difference in the projected number of days of heat stress between the daily and sub-daily methods of calculation for the 2010s compared to the 2090s. The increased T towards the end of this century increased the number of days when THI_d_ > THI_thr_, equalizing the number of days when THI_max_ > THI_thr_ (Fig 4). However, even in this situation the severity of heat stress, which is taken into account only in the case of the sub-daily estimation methods, increases AML (Fig 3).

The sub-daily method [8] is sensitive because it can detect days with relatively small heat stress loads, when the overall THI is below the threshold. This approach can quantify the load of heat stress rather than the average heat stress in estimating the extent and cumulative severity of heat stress within days. Thus, it is important in regions with temperate climates to quantify animal responses at acute stressful events, such as summer heat waves, because these responses will depend on the magnitude and duration of the heat wave [62]. Therefore, the combination of daily and sub-daily methods was used to represent a more biologically appropriate way of estimating milk loss from dairy cows due to heat stress. Indeed, the mixed “heat wave” methods (M5 and M6) are closer to current estimation of milk loses during heat waves [23] and probably provided a more realistic outcome for the future. The adequacy of the heat wave-based mixed method is also supported by the fact that its results match with that of the ensemble mean of all investigated methods.

In conclusion, we have developed a modelling framework to estimate potential effects of climate change on milk production of pasture-based dairy cattle using the UK as an example. We estimated relatively low AML that can be mitigated by implementing current practices for heat stress relief of cows on pasture. However, we detected specific regions of current dairy farming importance, where AML were projected to reach 17% of current annual milk yield in extreme years due to an increased frequency, duration and severity of heat waves. For these regions, the application of sophisticated technologies should be implemented to reduce projected losses. The choice of different THI threshold values made a large difference to projected milk loss. This observation alone emphasises the need for more intensive research seeking to determine the most biologically relevant THI_thr_ values of milk loss estimation methods and exploring the factors that influence this parameter. While this remains a challenging and complex issue [23], the approaches used in the present study provide a plausible solution that can be used in future climate change impact studies on pasture based dairy systems.

## Supporting information

S1 Text Milk loss estimation methods used in the study. (DOCX)
