# Supplementary material for: Spatially explicit estimation of heat stress-related impacts of climate change on the milk production of dairy cows in the United Kingdom
Source: PLoS One. 2018 May 8;13(5):e0197076. doi: 10.1371/journal.pone.0197076 (PMC5940184; doi:10.1371/journal.pone.0197076)
Supplement: S1 Text — (DOCX) [file pone.0197076.s001.docx]

**Supporting information**

**Milk loss estimation methods used in the study**

**M1: Yousef/St-Pierre (sub-daily step) method**

According to St-Pierre *et al.* (2003) THI follows an idealised sine curve during the day having a maximum (THI_max_) at 4 pm and a minimum (THI_min_) at 4 am [1]. Both THI_max_ and THI_min_ can be calculated with the equation proposed by Yousef (1985) using the maximum, minimum and dew point temperature (T_max_, T_min_, T_dew_, respectively) [2].

${THI}_{min}=T_{min}+0.36\times T_{dew}+41.2$ (1)

${THI}_{max}=T_{max}+0.36\times T_{dew}+41.2$ (2)

According to Schreck and Cook (2016), the diurnal variation of T_dew_ is relatively small [3]. During summer it is usually around ± 0.5 °C in Arkansas, USA. Based on our analysis of UK weather data (ERA Interim Database [4]), the expected value of the diurnal change of T_dew_ is ±1.5 °C around the mean during the summer. Despite this, T_dew_ was considered to be a daily constant which can be calculated by using the Magnus formula revised by Alduchov and Eskridge (1996) [5].

$T_{\mathrm{dew}}=\frac{B_{1}\times\left( \ln\left( \frac{\mathrm{RH}_{\mathrm{mean}}}{100} \right)+\frac{A_{1}\times T_{\mathrm{mean}}}{B_{1}+T_{\mathrm{mean}}} \right)}{A_{1}-ln\left( \frac{\mathrm{RH}_{\mathrm{mean}}}{100} \right)-\frac{A_{1}{\times T}_{\mathrm{mean}}}{B_{1}+T_{\mathrm{mean}}}}$ (3)

Where T_mean_ and RH_mean_ is the daily mean temperature and relative humidity, respectively, while A_1_ and B_1_ are constants with a value of 17.625 (unitless) and 243.04 (°C), respectively.

The actual time (t_1_) when THI exceeds THI_thr_ can be calculated by inverting the sine function that describes the daily course of THI resulting in the following formula.

$t_{1}=12\times\frac{\arcsin\left( \frac{\mathrm{THI}_{\max}+\mathrm{THI}_{\min}-{2\times THI}_{\mathrm{thr}}}{\mathrm{THI}_{\max}-\mathrm{THI}_{\min}} \right)}{\pi}+22$ (4)

Using the symmetry characteristics of the sine function the time (t_2_) when THI drops below THI_thr_ can be easily calculated. The value of 22 simply reflects the fact that the THI(t) sine curve that has its minimum and maximum at 04:00 and 16:00 intersects the horizontal (t) axes at 22:00. According to this method dairy cows are experiencing heat stress between t_1_ and t_2_. The amount of milk loss per cow (ML_1_, kg) is proportional to the duration (D = t_2_ - t_1_) of this period [8], and can be calculated with the following equation:

${ML}_{1}=0.0695\times\left( {THI}_{max}-{THI}_{thr} \right)^{2}\times D$ (5)

It has to be noted, that setting T_dew_ as a daily constant results in an underestimation of THI_max_, consequently the above equation tends to give a lower approximation of the daily milk loss, and therefore our milk loss estimates can be considered conservative.

**M2: NRC/St-Pierre (sub-daily step) method**

This method differs from the M1 method only in the way of calculating THI. During a day, temperature and relative humidity assumed to be counter-cyclical. Consequently, the minimum THI can be calculated using the minimum temperature and maximum relative humidity (RH_max_), while the maximum THI can be calculated using the maximum temperature and the minimum relative humidity (RH_min_). Both THI_max_ and THI_min_ are calculated with the equation proposed by the National Research Council [22].

${THI}_{max}=1.8\times T_{max}+32-\left( 0.55-0.0055\times{RH}_{min} \right)\times\left( 1.8\times T_{max}-26 \right)$ (6)

${THI}_{min}=1.8\times T_{min}+32-\left( 0.55-0.0055\times{RH}_{max} \right)\times\left( 1.8\times T_{min}-26 \right)$ (7)

Since T_dew_ was approximated as a daily constant, RH_min_ and RH_max_ could be approximated from the daily mean relative humidity by rearranging the Magnus formula for RH and substitute T_dew_, T_max_ and T_min_, as follows:

$\mathrm{RH}_{\min}=100\times exp\left( \frac{A_{1}{\times T}_{\mathrm{dew}}-\frac{A_{1}\times T_{\max}}{B_{1}+T_{\max}}\times\left( T_{\mathrm{dew}}+B_{1} \right)}{B_{1}+T_{\mathrm{dew}}} \right)$ (8)

$\mathrm{RH}_{\max}=100\times exp\left( \frac{A_{1}\times T_{\mathrm{dew}}-\frac{A_{1}{\times T}_{\min}}{B_{1}+T_{\min}}\times\left( T_{\mathrm{dew}}+B_{1} \right)}{B_{1}+T_{\mathrm{dew}}} \right)$ (9)

After calculating THI_max_ and THI_min_ the duration of the stress period (D) and the associated amount of milk loss was calculated similarly to M1.

**M3: Yousef/Bohmanova (daily step) method**

Using the daily mean temperature and dew point temperature, the daily effective temperature and humidity index (THI_d_) could be calculated with the formula proposed by Yousef (1985) [2]:

${THI}_{d}=T_{mean}+0.36\times T_{dew}+41.2$ (10)

Based on the study of Bohmanova et al (2007) the daily milk loss of dairy cows can be estimated with the following equation [6] using THI_d_.

${ML}_{3}=max\left( {THI}_{d}-{THI}_{thr},0 \right)\times0.37$ (11)

Where the max() function returns the maxima of its arguments.

**M4: NRC/Bohmanova (daily step) method**

The fourth method applies the NRC formula [7] for calculating the daily effective THI value using the daily mean temperature and relative humidity. In this case the empirical coefficient in the milk loss function proposed by Bohmanova *et al*. (2007) is 0.39 [6].

${THI}_{d}=1.8\times T_{mean}+32-\left( 0.55-0.0055\times{RH}_{mean} \right)\times\left( 1.8\times T_{mean}-26 \right)$ (12)

${ML}_{4}=max\left( {THI}_{d}-{THI}_{thr},0 \right)\times0.39$ (13)

**M5: Yousef/Heat wave-based mixed method**

The fifth method applies the Yousef formula (see M1) for calculating the THI value. Then it applies a mixed formula to account for the capacity of dairy cows to avoid heat stress and therefore milk losses in shorter periods of heat stresses risk [8,9]. Thus, on days of heat waves the St-Pierre method (see M1) is applied and on other days Bohmanova’s equation (see M3) is used to calculate milk loss.

**M6: NRC/Heat wave-based mixed method**

The sixth method differs from the M5 method in that the THI calculation uses the NRC formula (see M2).

**References**

1. St-Pierre NR, Cobanov B, Schnitkey G. Economic losses from heat stress by US livestock industries. J Dairy Sci. 2003;86: 52-77.
2. Yousef MK. Stress physiology in livestock. Volume 1, Basic principles. CRC Press, Boca Raton, FL, USA; 1985.
3. Schreck M-B, Cook KR. [Internet]. Dewpoint Climatology For The Wichita KS Forecast Area. 2016. [cited 2016 Sep 23]. Available from: https://www.weather.gov/ict/dewclimo1
4. Dee DP, Uppala SM, Simmons AJ, Berrisford P, Poli P, Kobayashi S, Andrae U, et al. The ERA-Interim reanalysis: configuration and performance of the data assimilation system. Q J Roy Meteor Soc. 2011;137: 553-597.
5. Alduchov OA, Eskridge RE. Improved Magnus form approximation of saturation vapor pressure. J Appl Meteorol. 1996;35: 601-609.
6. Bohmanova J, Misztal I, Cole JB. Temperature-Humidity indices as indicators of milk production losses due to heat stress. J Dairy Sci. 2007;90: 1947-1956.
7. National Research Council. A guide to environmental research on animals. National Academy Press. Washington DC; 1971.
8. Tucker CB, Rogers AR, Schütz KE. Effect of solar radiation on dairy cattle behaviour, use of shade and body temperature in a pasture-based system. Appl Anim Behav Sci. 2008;109: 141-154.
9. Schütz KE, Rogers AR, Cox NR, Webster JR, Tucker CB. Dairy cattle prefer shade over sprinklers: Effects on behavior and physiology. J Dairy Sci. 2011;94: 273-283.
